# Intra-Varietal Diversity and Its Contribution to Wheat Evolution, Domestication, and Improvement in Wheat

**DOI:** 10.3390/ijms241210217

**Published:** 2023-06-16

**Authors:** Tianbao Li, Chuizheng Kong, Pingchuan Deng, Chengdao Li, Guangyao Zhao, Hongjie Li, Lifeng Gao, Dangqun Cui, Jizeng Jia

**Affiliations:** 1 The College of Agronomy, State Key Laboratory of Wheat and Maize Crop Science, Henan Agricultural University, 63 Nongye Road, Zhengzhou 450002, China; 2 Institute of Crop Sciences, Chinese Academy of Agricultural Sciences, Beijing 100081, China; 3 State Key Laboratory of Crop Stress Biology in Arid Areas, College of Agronomy, Northwest A&F University, Xianyang 712100, China; 4 Western Barley Genetics Alliance, College of Science, Health, Engineering and Education, Murdoch University, Murdoch, WA 6150, Australia

**Keywords:** wheat genome, gene duplication, homolog diversity, subgenome divergence, transposable elements

## Abstract

Crop genetic diversity is essential for adaptation and productivity in agriculture. A previous study revealed that poor allele diversity in wheat commercial cultivars is a major barrier to its further improvement. Homologs within a variety, including paralogs and orthologs in polyploid, account for a large part of the total genes of a species. Homolog diversity, intra-varietal diversity (IVD), and their functions have not been elucidated. Common wheat, an important food crop, is a hexaploid species with three subgenomes. This study analyzed the sequence, expression, and functional diversity of homologous genes in common wheat based on high-quality reference genomes of two representative varieties, a modern commercial variety Aikang 58 (AK58) and a landrace Chinese Spring (CS). A total of 85,908 homologous genes, accounting for 71.9% of all wheat genes, including inparalogs (IPs), outparalogs (OPs), and single-copy orthologs (SORs), were identified, suggesting that homologs are an important part of the wheat genome. The levels of sequence, expression, and functional variation in OPs and SORs were higher than that of IPs, which indicates that polyploids have more homologous diversity than diploids. Expansion genes, a specific type of OPs, made a great contribution to crop evolution and adaptation and endowed crop with special characteristics. Almost all agronomically important genes were from OPs and SORs, demonstrating their essential functions for polyploid evolution, domestication, and improvement. Our results suggest that IVD analysis is a novel approach for evaluating intra-genomic variations, and exploitation of IVD might be a new road for plant breeding, especially for polyploid crops, such as wheat.

## 1. Introduction

Genetic diversity has an essential impact on the adaptation and productivity of crops in agriculture [1]. Analysis of genetic diversity often focuses on allelic diversity among accessions. Allelic diversity analysis has been widely used in genetics, genomics, germplasm research, and breeding, with significant achievements in understanding how genetic diversity has driven crop domestication and genetic improvement. Common wheat (*Triticum aestivum* L.) is a hexaploid crop species containing subgenomes AA, BB, and DD, formed by natural hybridizations of three diploid ancestors [2]. Previous studies revealed poor diversity in common wheat, which resulted from polyploidization and domestication. It has been a major barrier to further improvement of wheat [3,4,5].

Homologs within an individual include paralogs and orthologs in polyploids, which constitute a large component of the complete sets of genes in a species such as wheat. Paralogs are a set of duplicated genes derived from a common ancestral gene in the genome [6]. Gene duplication is a major force for evolution and the basis for generating new genes and alleles for organisms to maintain the stability of their genetic system and adapt to environmental changes [7,8]. Polyploid species have a greater proportion of homologs, comprising paralogs and orthologs. Systematic analyses of paralogs have been undertaken on diploid species [9,10]. However, no studies have comprehensively analyzed the pattern and diversity of paralogous genes in polyploids despite more than half of the plant species being polyploidy [11,12]. A considerable proportion of genes are duplicated in wheat, underpinning wheat evolution, domestication, and improvement. Homologs, such as genes *ZIP4* (*Ph1*) and *CENH3*, are essential for chromosome pairing, mitosis, and meiosis [13]. *Btr1* for spike brittleness is a key gene in wheat domestication. The dwarfing gene *Rht1* and photoperiod response gene *Ppd1* contribute significantly to the ‘Green Revolution’.

Homolog diversity or IVD refers to variation in sequence, expression, and function within a variety. Homologs coordinately confer plant growth, development, reproduction, and adaptation. Therefore, IVD analysis (IVDA) is expected to be an important and new area of diversity study.

Here, we carried out an IVDA of Aikang 58 (AK58), a modern leading wheat variety in China with high yield and diverse environmental adaptability, and Chinese Spring (CS), a landrace, both with high-quality reference genomes available, to reveal the sequence, expression, and functional diversity of homologs in the common wheat genome. We also evaluated how homolog diversity has shaped wheat evolution, domestication, and improvement.

## 2. Results and Discussion

**Figure 1:**
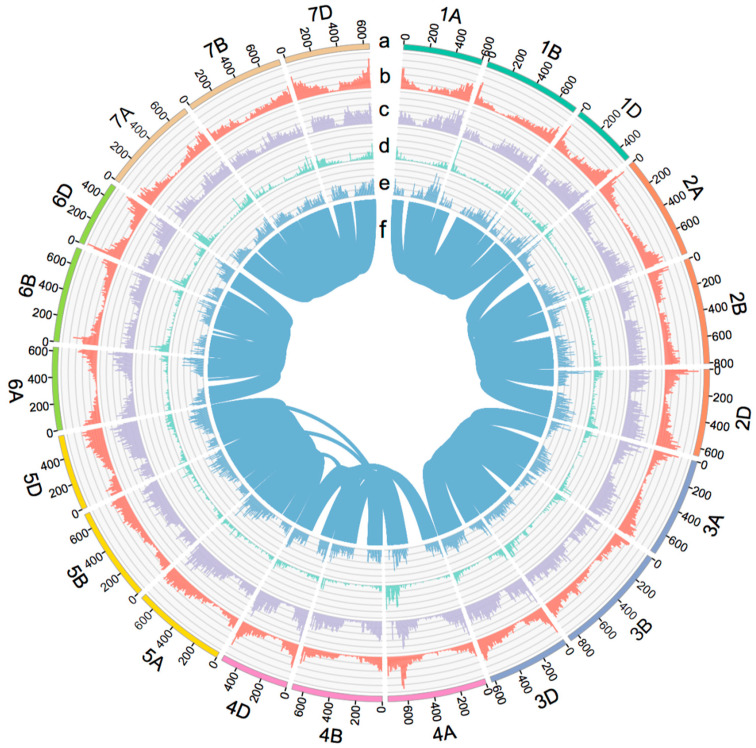
Circo’s plot display of the three homolog types on the wheat AK58 genome. The 21 chromosomes with names and sizes (a). Distribution of all the 119,448 HC genes (b), SORs (c), IPs (d), OPs (e), and collinear gene pairs of OPs among subgenomes (f).

A total of 119,448 high-confidence genes were annotated in the AK58 genome, including orthologs, paralogs, and singletons. We divided them into four types based on the distribution of homologous genes in the subgenomes: (1) IPs (inparalogs), duplicated genes in a single subgenome; (2) dyads, duplicated genes in two subgenomes; (3) triplets, duplicated genes in all the three subgenomes, of which OPs (outparalogs) are paralogous genes with collinearity between subgenomes in triplets; and (4) single-copy orthologs (SORs) with one copy in each subgenome. There were 12,762 gene families containing 44,568 paralogous genes and 13,780 gene families with 41,340 SOR genes, accounting for 71.9% (37.3% and 34.6%, respectively) of the total genes in common wheat (Table 1, Figure S1). The triplet paralogs had 4314 OP families based on their collinearities, including 13,131 paralogous genes. The proportions of each type of homologous genes in CS closely resembled those in AK58 (Table 1). Since IPs, OPs, and SORs accounted for most of the duplicated genes, we focused on analyzing these genes in the following text. These homologs were widely distributed in the genome (Figure 1). These results indicated that the homologs/duplication genes are an important part of the wheat genome.

### 2.1. IVD in Their Sequence, Expression, and Function

#### 2.1.1. Efficiency of IVD Detection

To test whether IVD was detected correctly and efficiently, we selected 16 gene ontology (GO) terms with the most homologs (from 312 to 1040 genes) to analyze IVD. Homologs conferring signal transduction, phosphorylation, and response to biotic and/or abiotic stress had a high diversity, while those conferring translation, abscisic acid-activated signaling pathway, and embryo development ending in seed dormancy had low diversity (Table S1). These results are consistent with the general gene diversity detected with the allele diversity analysis [14], suggesting that our method of IDA is an efficient approach for IVDA.

#### 2.1.2. Increased Sequence Diversity of OPs

**Figure 2:**
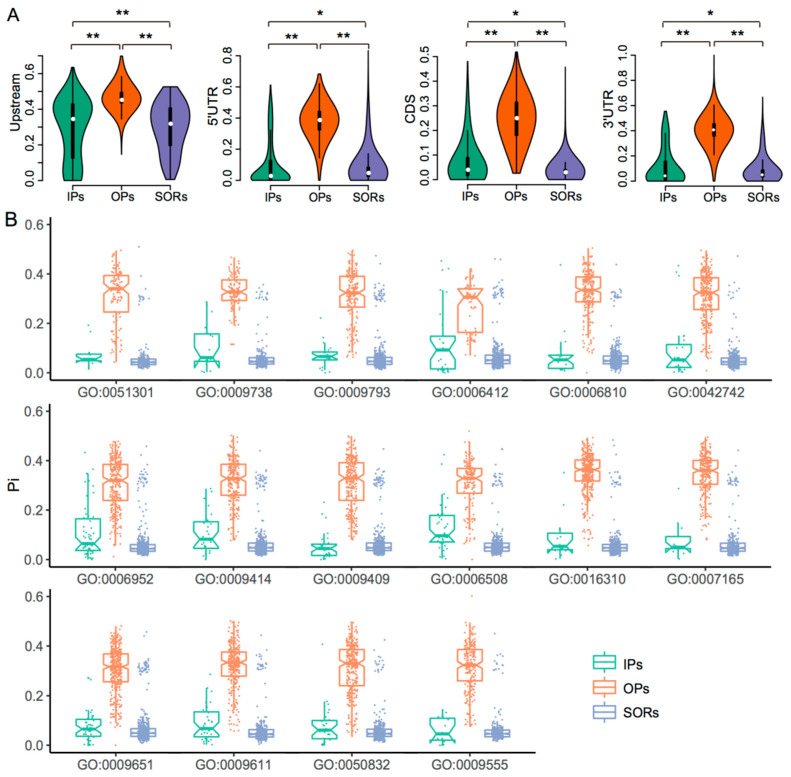
Homolog diversity of the AK58 genome. (**A**) Nucleotide diversity of homologs IPs, OPs, and SORs in regions of upstream 2Kb, 5′UTR, CDS, and 3′UTR (Wilcoxon signed-rank test, *: *p* < 0.05, **: *p* < 0.01). (**B**) Boxplots showing the homolog diversity for homologs IPs, OPs, and SORs based on the 16 GO terms. *Y*-axis, π values in the range of 0~0.6. *X*-axis, the GO terms. Each term corresponds to three types of homolog groups: Ips, Ops, and SORs. The mean π values of Ops are significantly higher than that of Ips and SORs for corresponding GO terms.

We analyzed the DNA nucleotide diversity of four regions with a gene—2 Kb upstream, 5′ UTR, coding sequence, and 3′ UTR of 8957 IPs and 13,131 OPs in the AK58 and CS genomes—for comparison of SORs in corresponding regions. The homologs had an average nucleotide diversity (π) of 0.2219, with the greatest diversity in OPs (0.3719), similar to SORs and IPs (Table 2, Figure 2A). We compared the diversity among OPs, IPs, and SORs for the 16 GO terms, generating similar results with nucleotide diversity (Figure 2B). A similar pattern of nucleotide diversity was also observed in the CS genome (Figure S2A), indicating that OPs have higher diversity in common wheat. Of the four regions, the 2 Kb upstream region had the greatest diversity in contrast with the lowest for CDS. The OPs had similar sequence diversity in the above intervals for the three subgenomes, indicating similar paralog diversity (Figure S2B) and, thus, similar evolutionary experiences. The similar homolog diversity of the three subgenomes may contribute equally to wheat evolution, domestication, and improvement. In contrast, an allelic diversity analysis suggested that the diversity of the three subgenomes is often different, with greater diversity for the subgenome B than subgenome D. Noteworthy, the IVD detected here is much higher than the allele diversity (π = 0.0015, πA = 0.0017, πB  =  0.0025, πD  =  0.0002) [4], suggesting that IVD has a great potential to be exploited for wheat improvement.

The OP pairs had a *Ks* of 1.114 with dispersed distribution (Figure S2C). The three subgenomes had similar *Ks* values. These results suggest that the OPs occurred 60 MYA when the three subgenomes diverged, and it may have lasted for a long period. In contrast, the IP pairs only had a *Ks* of 0.094, significantly lower than the OP pairs (Table S2, Data S1 and S2), suggesting that the IPs occurred after the divergence of subgenomes. Higher diversity was associated with early occurrence, possibly due to their accumulation over a long period.

**Figure 3:**
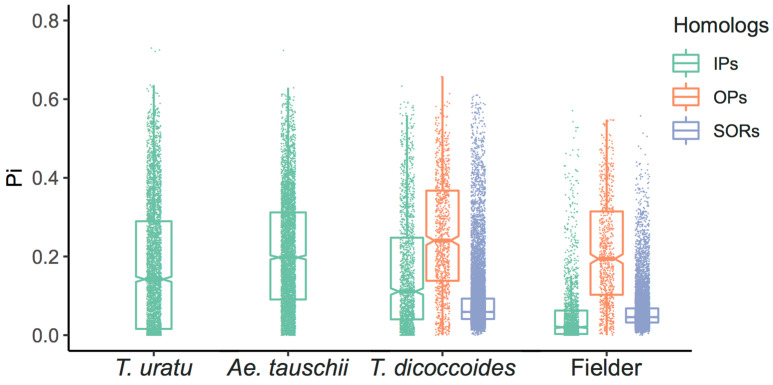
Homolog diversity of *T. uratu*, *Ae. Tauschii*, *T. dicoccoides*, and wheat cultivar Fielder genome. Boxplots showing the gene body diversity for homologs IPs, OPs, and SORs in the four genomes. *Y*-axis, π values in the range of 0~0.8. *X*-axis, the wheat varieties. There are only IPs in the *T. uratu* and *Ae. tauschii* genomes, *T. dicoccoides,* and Fielder genomes correspond to the three types of homolog groups, IPs, OPs, and SORs.

To detect the genetic diversity of different homologs during wheat evolution, we adopted the same approach to homologs identification in *Triticum uratu*, *Aegilops tauschii*, the diploid ancestor, wild emmer *Triticum dicoccoides* and wheat cultivar Fielder, respectively, and then analyzed the sequence diversity of upstream 2Kb, CDS and gene body of these homologous groups. Consistent with the situations of AK58 and CS, the diversity of OPs was the highest in the three homologs (Table 3, Figure 3), much higher than that of the IPs. It is worth noting that the diversity of IPs, OPs, or SORs decreased from the diploid progenitor species to the wild tetraploid and then hexaploid cultivars. More work is needed to understand this phenomenon in the future.

#### 2.1.3. Increased Expression Diversity of OPs and SORs

Expression variations often cause homolog subfunctionalization [15,16]. We analyzed the expression variation of homologs using RNA sequencing and expression quantification on 44 AK58 sample sets, including roots, stems, leaves, flowers, seeds, and tissues under different treatment conditions at different growth stages. We examined homolog variations in expression, co-expression, and tissue-specific expression.

**Figure 4:**
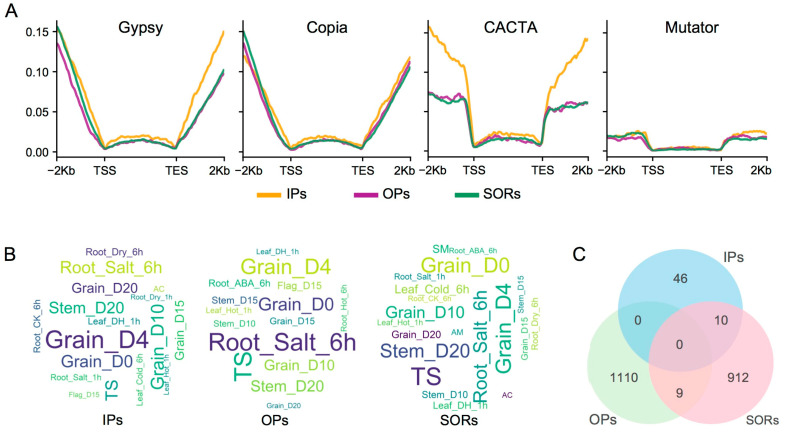
TEs levels, tissue-specific expression, and GO enrichment of homologs in AK58. (**A**) Density of LTR TEs (Gypsy and Copia) and DNA TEs (CACTA and Mutator) around homologs. TSS and TES indicate the transcription start site and transcription end site, respectively. (**B**) Tissue-specific expressions of IPs, OPs, and SORs. The bigger the font size, the higher the expression levels. (**C**) The number of enriched GO terms specific to and shared by IPs, OPs, and SORs. Figures in the overlapping areas are common GO terms.

OPs and SORs had the highest expression abundance in the three homolog types, in comparison with the lowest IPs (Figure S2D), suggesting that the divergence of OPs and SORs may have more functional variations in wheat than that of IPs. The low IP abundance may be associated with a higher Gypsy and CACTA content in the flanking regions (Figure 4A), which might be associated with Epi-modification. High CpG and CHG methylation often inhibit expression, while CHH methylation often activates expression.

The AK58 genome-wide genes can be divided into 84 co-expression modules. A total of 3106 OP families (or 72.0% of OP families) were diverged in co-expression modules, followed by 32.7% of the SOR families and 20.6% of the IP families, indicating that homologous expression diversity of subgenomes significantly differs in co-expression (Table S3). The higher expression diversity of OPs was consistent with the higher sequence diversity in the 2 Kb upstream regions, as the promoter regions play key roles in regulating gene expression.

IPs, OPs, and SORs had tissue-specific expression patterns. IPs were expressed most actively in early seed development. The expression of OPs peaked in roots under salt stress and in anthers. SORs had the highest expression in the stem and anther (Figure 4B). Different expression patterns suggest functional divergence of OPs, SORs, and IPs.

#### 2.1.4. Functional Divergence of Homologs

Sequence and expression diversity often generate functional changes in homologs [17,18]. Candidate neo-functionalization and sub-functionalization can occur in homologs, as detected by GO term and expression change, respectively [19,20]. We examined the homolog diversity from homolog-specific genes, GO enrichment, and family diversity. We obtained 7380 GO terms with 77,233 genes (65.7%) in the AK58 genome (Data S3, Figure S3A). Most GO terms are shared by different homologous groups. However, the SORs and OPs had 1699 (23.0%) and 221 (3.0%) specific GO terms, respectively, much more than the IPs (2 GO terms) (Figure S3B). In the GO enrichment analysis, 46, 1110, and 912 specifically enriched GO terms were detected in IPs, Ops, and SORs, respectively. IPs and SORs shared ten GO enrichments, OPs and SORs shared nine, but IPs and OPs shared none (Figure 4C). More OPs played roles in small molecular binding and catalytic activity. More IPs had functions related to reverse regulation mechanisms of protein and coenzyme metabolic/catabolic processes, while more SORs were related to nucleic acid metabolism and gene expression (Data S4, Figure S3C). These results suggest that the biological functions of homologs differ in common wheat. Consistent with the pattern of GO terms, the PO enrichment analysis revealed that different homologs had various PO functions (Data S5, Figure S3D). The OPs were significantly enriched in 151 PO terms. In comparison, SORs were enriched in 163 PO terms, with 45 overlapping the OPs. IPs were enriched in only three PO terms (hilum lateral roots and lateral root tips). Among the top 20 PO terms, OPs were related to anatomy and structure development of root, shoot, leaf, and spike, while SORs were mostly associated with shoot development (Data S5). The PFAM diversity analysis revealed that OPs had the greatest diversity (Data S6), consistent with the GO analysis.

### 2.2. Important Role of IVD in Wheat Evolution, Domestication, and Improvement

Generally, duplicated genes are prone to be lost or subject to pseudogenization. However, over 40,000 duplicated genes were stable in the wheat genome, suggesting that duplicated genes have important functions. Therefore, we examined how IVD contributed to wheat evolution, domestication, and improvement.

#### 2.2.1. Impacts of IVD on Wheat Evolution

**Figure 5:**
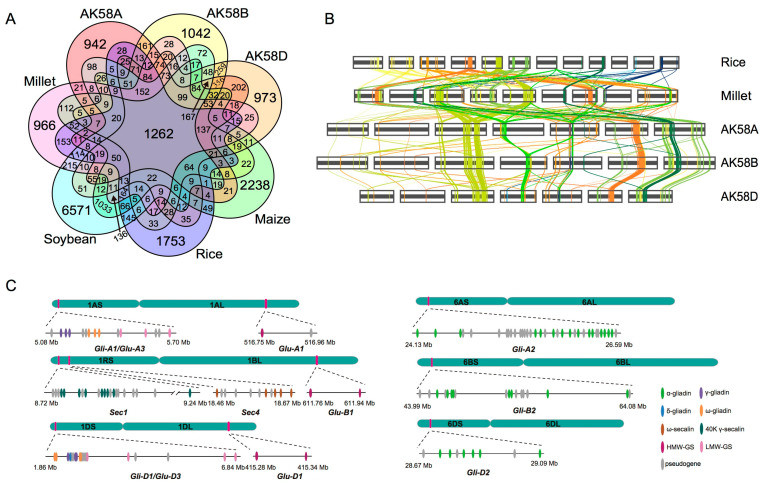
Common and specific paralogs in the AK58 genome. (**A**) Venn plot showing numbers of common and specific paralog families across wheat, rice, maize, millet, and soybean genomes. The high percentage of common paralogs indicates duplication before the divergence of dicotyledon and monocotyledon. (**B**) Collinearity of WGDPGs among the rice, millet, and AK58 subgenomes (AK58A, B, and D). (**C**) The distribution of storage protein genes on chromosomes 1A, 1B, 1D, and 6A, 6B, 6D of AK58.

Whole-genome duplication (WGD) and polyploidization are important in crop evolution [21]. To analyze the effect of whole-genome duplication genes (WGDGs) on wheat evolution of wheat and other major crops (i.e., rice, maize millet, and soybean), we compared the WGDG patterns of seven genomes/subgenomes. The five crop species had 1262 common WGDPGs (Figure 5A). These common duplication genes are mainly transcription factors (TF), signal transduction, and hormone-related genes. The functions are mainly involved in the development and abiotic stress resistance. For example, Ca(^2+^)-dependent protein kinases (CDPK) play an important function in the Ca(^2+^)-mediated signal transduction. Twenty-nine CDPK genes have been identified in the rice genome. OsCPK10 mediates drought tolerance and blast disease resistance, OsCPK12 is involved in salt stress tolerance and blast disease resistance, Oscpk7 confers tolerance to both salt and drought stresses, and OsCPK26 is involved in pollen development. These results suggest that the duplication genes in a family have divergence in their functions during the long period of evolution. However, the retention of common duplicated genes in different species demonstrates that the genes for plant development and tolerance to abiotic stresses are relatively conserved, and more genes are required to ensure crop development and adaptation to various environments. We detected 504 Gramineae WGDPGs with collinearity (Data S7, Figure 5B) and other specific WGDGs (Data S8, Figure 5A), as most grasses species have unique gene families ranging from 1000 to 2000. The numbers of unique WGDGs in the three wheat subgenomes were similar, indicating that the three subgenomes are closely related to Gramineae.

The species-specific homologs in subgenomes A, B, and D were significantly enriched in the regulation of biosynthetic processes, nucleic acid binding, and negative regulation of coenzyme metabolic processes, respectively (Data S9). The specific WGDGs of subgenomes A, B, and D had 45, 48, and 33 enriched KEGG biological pathways, respectively, with only a few common pathways, indicating their functional divergence (Data S10, Figure S4). All these homologs contribute to the evolution of wheat and other species, which endow common and specific characteristics to the species.

Gene expansion is a specific type of homolog that often contributes to specific species traits [22,23]. We defined a gene copy number of five or more in a single genome as an expansion family and found 650 expansion gene families, including 6636 genes distributed on wheat chromosomes, followed a similar pattern to the total gene distribution in the wheat subgenomes (Figure S5). The expanded gene families were 31.6%, 6.4%, and 8.3% more than that in rice (494), maize (611), and millet (600), respectively (Data S11-1). The expansion genes accounted for 16.8% of the total genes in wheat and 10.1% in rice, suggesting the significant contribution of gene expansion to the gene number in crop genomes. The seven studied genomes shared 159 common expansion families with 2334 genes. On average, 15.3 gene copies occurred in each common family, 50% higher than the total expanding genes, suggesting that ancient expansion families may have more gene copies. The nucleotide diversity (Pi) of the expansion genes was 0.47 on average for the four regions, upstream, 5′UTR, 3′UTR, and CDS, of the gene, which is 27% higher than the total OPs. This indicates that more diversity occurred in expansion genes. The GO enrichment analysis showed that these genes had diverse functions (Figure S6), including 1268 GO terms. The top five functions were catalytic activity, small molecule binding, drug binding, nucleoside phosphate binding, and carbohydrate derivative binding. We collected the genes in rice (114 families and 488 genes) to further analyze the functions of common expansion genes (Data S11-2). Their functions included resistance to biotic and abiotic stresses, plant growth and development, and seed quality, demonstrating that expansion genes are functionally diverse.

We compared the expansion genes between the Gramineae or the grass family, such as wheat, rice, maize, and millet, and *Leguminosae*, such as soybean. Eight grass-specific or dominant expansion families, including 172 genes on average, were detected (Table S4-1). The copy number of these genes expanded ranged from 8 to 92 in grass species but only 0 to 2 in soybean. Of the eight families, two families, Group 2 and Group 141, are involved in diseace resistance. Group 2 is a super family with 92 copy numbers, and some disease-resistance genes, such as *Pi36*, *OsPi304*, *Pita*, and *Pi-4a* for rice blast resistance, were identified. Group 141 is a big family with 11 copy numbers on average for disease resistance, such as *Pikp-2* for rice blast resistance. The other six families, Groups 34, 138, 162, 192, 215, and 252, are involved in plant development and resistance to abiotic stress. The common expanded biotic stress resistance families in the grass might be because grass species have similar diseases but are different from dicots. For example, *Fusarium graminearum* causes *Fusarium* head blight in wheat, barley, and rice, as well as stalk and ear rot in maize. It also causes similar plant phenotypes and/or developmental characteristics. Group 252 is a nitrate transporter-related family that positively regulates tiller number and grain yield. The grass families develop a similar nitrate transporter system, which is different from soybean, that has a nitrate fixation system. These expansion families may contribute to the divergence between monocots and dicots.

We then compared expansion gene families between wheat and other grass species, i.e., rice, maize, and millet. Totally 46 large families with copy numbers of more than 20 in any one of the above species were selected, and the total gene number is up to 1782 in wheat (Table S4-2). Noteworthy, of the 46 large families, 39 families (84.8%) showed more copy numbers in wheat than in other species. The total copy number of expansion families in wheat is 78% more than that in the other grass species (Table S4-2). Four families, Group 33, 35, 61, and 89, have copy numbers three folds over the other grass species, and fifteen families have copy numbers at least two-fold of the other grass species, indicating that these families are dominant expansion families. For example, there are 47, 29, and 32 copies in SubA, SubB, and SubD of wheat for Group 35, but only 10 copies in the other grass species on average. Of the wheat-specific expansion families, 23 (60.1%) families are involved in abiotic stress resistance, and 16 (42.1%) families, including 7 (18.4%) overlapped with abiotic stress resistance, are involved in biotic stress resistance. Eighteen (47.4%) families are associated with development-related genes, including 10 overlapped with biotic or abiotic stress resistance. The nucleotide diversity (Pi) of these families is as high as 0.5037, 35.6% higher than that of the OPs and even higher than the total expansions genes (Pi = 0.4721), suggesting that larger families might be more diverse. Group 2 is a supper family in the grass, with 105, 155, and 136 copies in SubA, SubB, and SubD, respectively. Five disease resistance genes have been identified in this family, including *NLS1*, the CC-NB-LRR-type *R* gene, in rice, and stem rust resistance gene *Sr22* in wheat [24]. In addition, there are seven members in Group 2 mapped in similar regions as the QTL conferring yellow rust resistance (Table S5). We propose that some of these members might be the candidate genes of yellow rust resistance. The large copy numbers will be a source for potential functional diversity.

We further analyzed species-specific expansion gene families (Data S11-3). Eleven specific expansion families comprising 197 genes were detected in Gramineae (Data S11-4). The GO enrichment analysis indicated their functions in carbohydrate derivative binding, cell death, defense response, ion binding, and stimulus-response. Their functions were reflected in several agronomically important traits, such as disease resistance, yield, plant architecture, and nitrogen use efficiency (Data S11-5). A number of 16, 25, and 28 GO terms were enriched for the three wheat subgenomes, all of which are different (Table S6, Figure S7), which suggests that these genes contribute to the functional divergence of the three wheat subgenomes. The TO enrichment analysis indicated that these genes confer functions in inflorescence branching, inflorescence shape, and plant embryo morphology, contributing greatly to plant phylogeny classification.

Wheat has the highest protein content over other major food crops, such as rice, maize, and millet. The exact number of gene-encoding proteins in wheat has been unclear due to the difficulty in assembling the high tandem duplication. However, using the high-quality AK58 genome, we detected eight expansion storage protein families, with approximately 80 copies in each subgenome (Figure 5C), compared to 22 gene copies in rice, 27 in millet and sorghum, 47 in maize, and 118 in soybean [25,26,27]. The grain protein content was significantly correlated with the copy number of the storage protein genes (*R* = 0.86, *p* < 0.03). These results suggest that the high protein contents of wheat and soybean are such as the result of the expansion of storage protein genes, and large and diverse expansion families contribute to wheat adaptation, making wheat a world crop.

Polyploidization and its subsequent diploidization are two important evolutionary processes in wheat evolution. Two genes, *CENH3* and *Ph1* (*ZIP4*), play key roles in these processes in hexaploid wheat. Notably, both of them are OPs.

**Figure 6:**
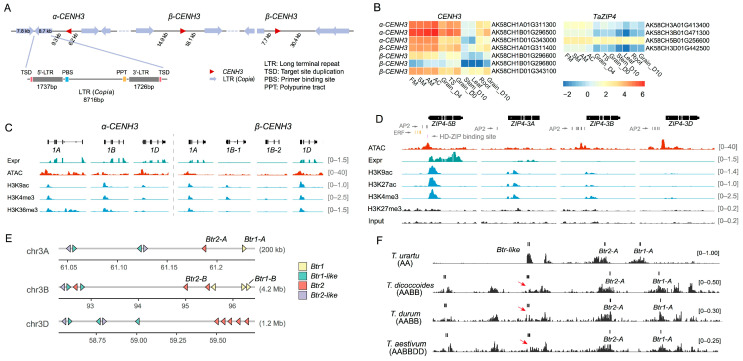
Expression, chromatin accessibility, and epigenetic modification of *CENH3*, *TaZIP4,* and *Btr* duplications. (**A**) Tandem duplications *CENH3* on chromosome 1B of AK58. *CENH3* patterns on 1B are conserved between hexaploid and tetraploid wheat. Similar TE families flank the *CENH3* duplications, resulting in non-homologous recombination. (**B**) Expression heatmap of *CENH3* and *TaZIP4* in AK58 tissues at different stages and conditions. Relatively, the expression levels of *α-CENH3* are higher than those of *β-CENH3*. Tissues are collected for leaf and root at the seedling stage; stem 10 days after anthesis; young spike at the spikelet meristem stage (SM), floret meristem stage (FM), anther primordia stage (AM), tetrads stage (TS), and anther connective stage (AC); developing grains four days (D4) and 10 days (D10) after anthesis. (**C**) Genomic tracks illustrating expression (Expr), chromatin accessibility (ATAC) and main active histone marks (H3K9ac, H3K4me3 and H3K36me3) of *CENH3*. (**D**) Genomic tracks illustrating chromatin accessibility (ATAC), expression (Expr), and binding of H3K9ac, H3K27ac, H3K4me3, H3K27me3 and their control (Input) of *TaZIP4*. Bars below the *TaZIP4* schematic diagrams indicate co-expressed TF binding sites within 1.5Kb promoters: AP2 (black), ERF (orange), and HD-ZIP (violet). (**E**) The location of *Btr1*, and *Btr2* and their like genes on chromosomes 3A, 3B, and 3D. (**F**) H3K27me3 modification of *Btr* and *Btr-like* OPs in *T. urartu* (AA), *T. dicoccoides* (AABB), *T. durum* (AABB) and *T. aestivum* (AABBDD) on chromosome 3A. The red arrows indicate the low enrichment or loss of H3K27me3. The H3K27me3 data are retrieved from NCBI BioProject (accession no. PRJNA609457). The *Btr* and *Btr-like* genes, are annotated manually.

The centromere is a key functional region of chromosomes, essential in genome polyploidization [28]. *CENH3* encodes a centromere-related protein associated with plant height [29,30]. Only one copy of *CENH3* was found in rice and maize, but two copies (*α-CENH3* and *β-CENH3*) were detected in diploid, tetraploid, and hexaploid wheat [31]. We confirmed the presence of two copies (*α-CENH3* and *β-CENH3*) on wheat chromosomes 1A and 1D. Furthermore, we found three copies in tandem duplication, one *α-CENH3* and two *β-CENH3*, on chromosome 1B of hexaploid and tetraploid wheat, including durum wheat (*T. durum*) [32] and wild emmer (*T. dicoccoides*) (Figure 6A). We detected similar TE families flanking these duplicated genes, which are likely generated from the non-homologous recombination. Variation in GO differed for *α-CENH3* and *β-CENH3*, with more GO terms assigned to *β-CENH3* (Table S7), suggesting divergent functions. Higher expression of *α-CENH3* was detected in subgenomes A, B and D, but *β-CENH3* only in A and D. (Figure 6B). We also found that higher gene expression was associated with chromatin accessibility (Figure 6C). All genes with high expression had high chromatin accessibility and active histone marks. In contrast, genes with lower expression were less or not accessible. The expression of *CENH3* varied between tissues, with the highest expression in FM, consistent with its chromosomal pairing function, and it was also detected in developing grains, leaves, and roots (Figure 6B). We mapped QTL for plant height, grain yield, grain number per spick, and thousand-grain weight (TGW) on the flanking regions of *CENH3*. Two mutant lines with missing variation in *α-CENH-1B* and *α-CENH-1D* had short plant height, confirming their function in plant stature. We also detected a stop gain mutation of *α-CENH-1A* in an AK58 EMS mutant line with the shriveled grain, which was confirmed by QTL mapping.

*ZIP4* (*Ph1*) promotes homologous pairing during meiosis [33,34], contributing to cytological diploidization. Four *ZIP4* copies, *ZIP4-3A*, *ZIP4-3B*, *ZIP4-3D*, and *ZIP4-5B,* were observed in common wheat. The first three are orthologs, and the *ZIP4-5B* sequence had a high similarity with *ZIP4-3B* (Figure S8), indicating that they are paralogs. *ZIP4-5B* was formed from the duplication of *ZIP4-3B* and then transferred to chromosome 5B.

The analysis of chromatin accessibility and histone modification revealed that *ZIP4-5B* had the highest accessibility near the upstream regions of the transcription start site, accompanied by higher transcription-activating modifications such as acetylated H3K9 and H3K27 than the other copies (Figure 6D). Although chromatin accessibilities were higher around *ZIP4-3D*, no enrichment of active histone marks was detected. Therefore, we proposed that chromatin accessibility and active modification activate *ZIP4-5B* expression (Figure 6B). In addition, Martin et al. [35] reported that *ZIP4-5B* was related to TGW and grain number per spike. We found multiple yield-related QTL in the region around *ZIP4-5B*, and several yield-related genes co-expressed with *ZIP4-5B* (Figure S9), including *DEP1* (Dense and Erect Panicle1), *GW2* (Grain Weight2), and *VRN1* (Vernalization1). In addition, 41 transcription factors (TFs) were co-expressed with *ZIP4* (Table S8), including those from *AP2*, *ERF*, and *HD-ZIP* TF families. The TF binding sites appeared within 1.5 Kb promoters upstream of *ZIP4-5B*, *ZIP4-3A*, *ZIP4-3B*, and *ZIP4-3D*. The AP2-class TF binding sites existed in all four copies of *ZIP4*, while *HD-ZIP* had a binding site in the accessible regions upstream *ZIP4-5B* and exclusively co-expressed with *ZIP4-5B*, which may explain its specific function. These results suggest that the *ZIP4* (*ZIP4-5B*) contributed greatly, combining with the other three copies to wheat post-polyploid diploidization, and may be a pleiotropic gene.

#### 2.2.2. Impact of IVD on Wheat Domestication and Improvement

Modern wheat experienced two major processes to shape its genome—domestication and improvement—with IVD significantly contributing to both processes. To reveal the contribution of IVD to wheat domestication and improvement, we collected the cloned 187 agronomic important genes and analyzed their IVD. These genes were associated with six agronomic traits and grouped into homologous types (Table 4). Except for the four singleton genes, 83, 99, and one gene belonged to the OPs, SOR, and IPs, accounting for 44.4%, 52.9%, and 0.5% of the 187 cloned genes, respectively. OPs and SORs in these agronomic genes amounted to 97.3%, much higher than the percentage of the total genes (45.7%). In contrast, IPs and singletons in the cloned genes were lower than the total genes (2.6% vs. 35.6%). These results are consistent with the diversity analysis above, where OPs and SORs have higher diversity while IPs are lower.

Next, we focused on analyzing the domestication gene *Btr1*. This gene confers brittle rachis brittleness, derived from its ancestral paralog *Btr-like* [36]. It has been indicated that domestication, intact or brittle rachis, and harvestable spikes are acquired by recessive loss-of-function mutations in *Btr1-A* (*TtBtr1-A*) and *Btr1-B* (*TtBtr1-B*) [37]. The domestication alleles were loss-of-function versions caused by a two base-pair deletion in *Btr1-A* and a 4 Kb insertion in *Btr1-B*. In the present study, we found that *Btr1* and *Btr2* and their associated-like genes were tandem duplications with three, five, and three copies located on chromosomes 3A, 3B, and 3D, respectively, for *Btr1*/*Btr1-like*, and three, four, and seven copies for *Btr2*/*Btr2-like* (Table S9, Figure 6E). Thus, both genes are OPs. We compared the sequences and expression variations between cultivars and their wild ancestor, *T. dicoccoides*. In addition to the known 2 bp deletion in *Btr1-A*, we discovered two variations in the *Btr1-like*. One is a 3 bp deletion in *Btr1_3-B*, and the other is an 8 bp deletion in *Btr1_2-B*. Significant expression divergences were present between *Btr1* and *Btr2* and among their duplication copies. Of the 25 *Btr* and *Btr-like* copies, the only active expression was *Btr1-like Btr1_2-B* (*TraesAK58CH3B01G071900LC*), and other copies had weak or non-expression (i.e., loss-of-function in AK58). It is worth noting that both prolamin and *Btr* genes were tandem duplication. However, most of the prolamin genes, *Gli-* and *Glu-*, were actively expressed, possibly due to artificial selection.

The expression divergence of *Btr* and *Btr-like* and their duplicate copies, may also be associated with epi-modification. The paralogs of *Btr* and *Btr-like* were DNA hypomethylated, with enriched levels of H3K27me3 in various tissues, including seedlings, leaves, stems, sheaths, flag leaves, and spikelets (Figure S10). This indicates that the gene expression was tissue-specific, consistent with specific expressions in the glume and lemma (Figure S11). The enriched H3K27me3 may inhibit expression outside these specific cells in spikes.

Further comparison of the H3K27me3 modification of *Btr*/*Btr-like* between *T. urartu*, wild emmer, durum wheat, and common wheat, showed that it was significantly decreased after the first polyploidization compared to *Btr* (Figure 6F and Figure S12). The *Btr* copies of H3K27me3 were expressed in spikes, while *Btr-like* was not (Figure S11). Therefore, we speculate that the variation in H3K27me3 modification is involved in the expression and functional divergences of *Btr* and *Btr-like* homologs.

## 3. Materials and Methods

### 3.1. Identification of Paralogs and Single-Copy Orthologs

We performed BLAST searches using NCBI BLAST v2.6.0+ to identify duplicate genes from all high-confidence genes in genomes of AK58 and other wheat varieties. We retained matches with identity ≥70% and e-values < 1 × 10^−5^ [38]. OrthoMCL v2.0.9 was adopted to cluster and construct gene families for all peptide sequences in the genomes [39]. We then identified IPs, dyads, triplets, and SORs by verifying the subgenomes in which these gene families exist. The OP families were identified by reciprocal BLAST of triplets among subgenomes A, B, and D, and then their collinearity was confirmed. MCScanX (https://sourceforge.net/projects/mcscanx, accessed on 9 April 2023) was used to construct genomic synteny blocks with parameters -s 3 -e 1 × 10^−5^ [40]. Circle plots were generated with Circos v0.69-6 [41]. The CS genome datasets were downloaded from URGI (https://wheat-urgi.versailles.inrae.fr, accessed on 30 April 2023), and the AK58 genome data were obtained from a recent study at https://ngdc.cncb.ac.cn/gwh/Assembly/9670/show (accessed on 30 April 2023). The diploid *T. uratu* [42], *Ae. tauschii* [43], tetraploid *T. dicoccoides* [44], and hexaploid cultivar Fielder [45] genomes were also used for identifying homologs following the same approach to detect the genetic diversity during wheat evolution.

### 3.2. Calculation of Ka/Ks Values and Homolog Diversity (HD)

The KaKs_Calculator v2.0 was used to estimate substitution rates at nonsynonymous/synonymous (*Ka*/*Ks*) sites of paralogs and SORs in AK58 using the MA method [46]. Gene sequence alignments were carried out with NCBI BLAST v2.6.0+, with a bidirectional best hit approach (e-value < 1 × 10^−5^, identity > 60%, and coverage > 60%) used for gene pairs in the *Ka*/*Ks* calculation. Only significant *Ka*/*Ks* values (*p* < 0.05) were used to ensure the reliability of the results. Other parameters were set to default values. These cutoffs were also used in the approach of CACTAs captured gene pairs. We removed the gene pairs with *Ks* ≥ 3 to be conservative. Such genes did not alter the *Ka*/*Ks* distribution even though they were excluded.

The homolog diversity analysis of gene families associated with important GO terms was carried out based on function annotations to identify homologous gene families. First, we searched for homologous gene families containing copies associated with 16 high-frequency annotated terms, retaining families with isolated copy numbers ≥ 2. Next, multiple sequence alignments were performed within each subfamily (copies with a target term) using MUSCLE v3.8.31 [47]. Finally, the homolog diversities were calculated with a Python v3.5.6 program, as assessed by visual inspection of boxplots. The boxplots were produced in the R package ggplot2 (https://ggplot2.tidyverse.org, accessed on 30 April 2023). The nucleotide diversity calculation of homologs was obtained with the index Pi [48].

(1) Pi=2XL×D×(D−1)

where D is the depth of sequence alignment, X is the number of variant sites, and L is effective alignment sites. The results are presented as medium, with the Wilcoxon rank sum test used to determine significant differences among gene regions (*p* < 0.05). All statistical analyses were performed using R version 3.6.3 (https://www.r-project.org, accessed on 30 April 2023).

A phylogenetic tree was constructed in MEGA5 using the neighbor-joining and maximum-likelihood methods with 1000 replicates in the bootstrap test [49].

### 3.3. Expression Divergence and Tissue Specificity

We used the fragments per kilobase of the exon model per million mapped fragments (FPKM) of leaf organs at the one-leaf stage to estimate gene expression levels. For each gene family, the extreme deviation and coefficient of variation (CV) were obtained from all FPKM values; and expression patterns were considered significantly diverged if CV > 0.6 and absolute log2FC ≥ 1 (*p* < 0.05). We restricted the co-expression analysis to gene families in which all copies had clear co-expression modules.

The tissue specificity expression index (τ) for each gene was determined by programming the following calculation in Python v3.5.6 [10]:

(2) $$\tau =\frac{\sum_{i=1}^{n}[1-X_{i}]}{n-1}$$

where $X_{i}$ is the value of each gene in the expression matrix using dispersion normalization, and n is the number of total samples in different organs. τ ranges from 0 to 1, where low τ values indicate broad expression and high τ values indicate tissue-specific expression. The RNA-seq dataset for CS was downloaded from the NCBI Gene Expression Omnibus database (https://www.ncbi.nlm.nih.gov/geo/query/acc.cgi?acc=GSE139019, accessed on 30 April 2023).

### 3.4. Gene Ontology Enrichment and KEGG Enrichment

We used biological process, molecular function, and cellular component ontologies released by the GO Consortium [50]. To determine whether paralogs have neo-functionalized among copies, we compared their GO functions and other ontologies. Paralogous or orthologous gene pairs are regarded as functions divergent if they have different GO Terms. ClusterProfiler v4.0 was used to determine GO enrichment and KEGG enrichment using Fisher’s Exact Test with Bonferroni correction [51]. Enriched terms or pathways at *p* < 0.05 were used for further downstream analysis steps.

### 3.5. Transposable Elements and Methylation Analysis

The genes were divided into three regions for analyzing TEs, GC content, and methylation: upstream 2 Kb, downstream 2 Kb, and gene body. The TE distribution on the above regions was programmed in Python code. The GC content was calculated in 200 bp windows. The bisulfite sequencing data were aligned to the AK58 genome using Bismark with bowtie2 v2.2.9 [52], adopting a paired-end mapping strategy. Following alignment, a Bismark extractor v0.18.1 was used to identify CpG, CHG, and CHH methylation. Subsequently, the methylation percentage was determined by comparing the ratio of T to C detected at any site. DeepTools v2.5.3 was used for the TE and methylation visualization [53]. The TE annotation and bisulfite sequencing datasets were obtained from a recent study [54].

### 3.6. Gene Expansion and WGDPGs Analysis

We relied on a sequence homology approach to identify gene expansion, further identifying WGDPGs by synteny blocks generated in WGD events. We completed the first all-versus-all peptide alignments using NCBI BLAST+ v2.6.0+ for all proteins in AK58, rice, maize, millet, and soybean genomes with BLASTP parameters -max_target_seqs of 1000 and a maximum e-value of 1 × 10^−5^. In the downstream analysis, genes with sequence identity and query coverage >70% were considered to construct gene families across different species, and the paired peptides cluster strategy used MCL v14-137 with the default settings [55]. Genomic synteny blocks within and between each genome were constructed using MCScanX (https://sourceforge.net/projects/mcscanx, accessed on 9 April 2023) with parameters -s 3, -e 1 × 10^−5^, respectively, to identify WGDPGs. Only paralogs in synteny blocks within and between genomes were considered common WGDPGs.

The rice genome Os-Nipponbare-Reference-IRGSP-1.0 and annotations were downloaded from the Rice Annotation Project Database (RAP-DB, https://rapdb.dna.affrc.go.jp/download/irgsp1.html, accessed on 30 April 2023). The maize genome Zm-B73-REFERENCE-GRAMENE-4.0 and annotations were downloaded from the Maize Genomics Resource (MGR, http://maize.uga.edu/MSU_func_download.shtml, accessed on 30 April 2023). The millet genome *Setaria italica* v2.0 and annotations were downloaded from Ensembl (https://plants.ensembl.org/Setaria_italica, accessed on 30 April 2023). The soybean genome *Glycine max* and annotations were downloaded from PlantGDB (http://plantgdb.org/XGDB/phplib/download.php?GDB=Gm, accessed on 30 April 2023).

## 4. Conclusions

The low level of allele diversity in common wheat, in particular commercial varieties, is found to be a main barrier to further wheat improvement. Intra-diversity analyses are a novel approach to examining genetic diversity within a genome. We carried out IDA to determine HD function in common wheat, representing the first intra-genome diversity analysis. Our results suggest that IDA is a powerful and efficient method for genomic research. Our IDA, based on the high-quality AK58 and CS genomes, showed that polyploids gain additional subgenome homolog diversity, i.e., OP and SOR, and thus have more genomic diversity than diploid species. Notably, subgenome homologs had a significantly higher proportion of agronomically important genes. Therefore, we proposed that subgenome homolog diversity is essential for polyploid heterosis. With the increasing availability of high-quality reference genomes and resequencing data, IDA could offer deep insights into genomic structure and function and become a routine tool in genomic research. Particularly, it could be used to evaluate crop germplasm diversity of individual accessions. The new capacity offered by IDA could consequently accelerate germplasm exploitation and utilization. This method could also be used to evaluate the diversity of gene families (e.g., *R* genes) and TFs within a genome, opening new frontiers in genomic analysis.

We found that higher diversity was revealed by homologs (π, 0.2219) than in previous reports by allelic diversity in wheat (π, 0.001, [14]), which is consistent with the divergence time. Homolog divergence usually takes millions of years, but allelic divergence occurred more recently, usually within the last few thousand years. We also found that the landrace CS had higher HD than the modern commercial variety AK58, possibly due to the long divergence time and selection in wheat breeding.

## Figures and Tables

**Table 1:** Homologs in the wheat genome.

	AK58			CS		
	Families	Genes	Percentage (%)	Families	Genes	Percentage (%)
Paralogs	12,762	44,568	37.3	11,360	40,560	37.9
IP	3693	8957	7.5	2772	6532	6.1
Dyad	2154	5649	4.7	1898	4748	4.4
Triplet	6915	29,962	25.1	6690	29,280	27.4
OP	4314	13,131	11.0	4299	13,068	12.2
SOR	13,780	41,340	34.6	13,393	40,179	37.6
Total		119,448	71.9		106,925	75.5

**Table 2:** The diversity (π) of IPs, OPs, and SORs in four regions of AK58 and CS.

Regions		Upstream 2 Kb	5’UTR	CDS	3’UTR	Average
IPs	AK58	0.2852	0.1002	0.0699	0.1105	0.1414
	CS	0.3477	0.1552	0.0791	0.1744	0.1891
	Average	0.3165	0.1277	0.0745	0.1424	0.1653
	*p*-Value	<2.2 × 10^−16^	<2.2 × 10^−16^	3.77 × 10^−8^	<2.2 × 10^−16^	0
OPs	AK58	0.4675	0.3774	0.2451	0.3965	0.3716
	CS	0.4720	0.3734	0.2467	0.3966	0.3722
	Average	0.4698	0.3754	0.2459	0.3965	0.3719
	*p*-Value	1.23 × 10^−6^	0.3791	0.3502	0.7882	0.3794
SORs	AK58	0.2974	0.0802	0.0343	0.0851	0.1243
	CS	0.3242	0.0822	0.0344	0.0913	0.133
	Average	0.3108	0.0812	0.0344	0.0882	0.1286
	*p*-Value	<2.2 × 10^−16^	<2.2 × 10^−16^	0.0017	<2.2 × 10^−16^	0
Average	AK58	0.3501	0.1859	0.1164	0.1973	0.2124
	CS	0.3813	0.2036	0.1200	0.2208	0.2314

**Table 3:** The average diversity (π) of Ips, Ops, and SORs in four species.

Regions		Ips	Ops	SORs	Average
	Upstream 2 Kb	0.4354	-	-	0.4354
*T. uratu*	CDS	0.1303	-	-	0.1303
	Gene body	0.1722	-	-	0.1722
	Upstream 2 Kb	0.5613	-	-	0.5613
*Ae. Tauschii*	CDS	0.2006	-	-	0.2006
	Gene body	0.2106	-	-	0.2106
	Upstream 2 Kb	0.4799	0.5559	0.4511	0.4956
*T. dicoccoides*	CDS	0.1277	0.1565	0.0428	0.1090
	Gene body	0.1587	0.2535	0.0871	0.1664
	Upstream 2 Kb	0.2758	0.5440	0.3898	0.4032
Fielder	CDS	0.0449	0.1209	0.0300	0.0653
	Gene body	0.0532	0.2120	0.0603	0.1085

**Table 4:** Homologous analysis of 187 cloned genes with agronomically important traits.

	OPs		SORs		IPs		Singletons		Total
	No.	Ratio	No.	Ratio	No.	Ratio	No.	Ratio	No.
Abiotic stress	18	0.217	32	0.323	0	0	0	0	50
Biotic stress	31	0.373	15	0.152	1	1	3	0.75	50
Architecture	3	0.036	6	0.061	0	0	0	0	9
Heading	3	0.036	6	0.061	0	0	0	0	9
Quality	6	0.072	9	0.091	0	0	1	0.25	16
Yield	19	0.229	27	0.273	0	0	0	0	46
Other	3	0.036	4	0.04	0	0	0	0	7
Total genes	83	1	99	1	1	1	4	1	187
Ratio 1		0.444		0.529		0.005		0.021	
Ratio 2		0.111		0.346		0.075		0.281	

Ratio 1: ratio of gene type to the 187 cloned genes. Ratio 2: ratio of gene type to the whole-genome genes.

## Data Availability

The data presented in this study are available in the Supplementary Materials.

## Supplementary Materials

The supporting information can be downloaded at: https://www.mdpi.com/article/10.3390/ijms241210217/s1. References [56,57,58,59,60,61] are cited in the Supplementary Materials.
