# Supplementary material for: Intra-Varietal Diversity and Its Contribution to Wheat Evolution, Domestication, and Improvement in Wheat
Source: Int J Mol Sci. 2023 Jun 16;24(12):10217. doi: 10.3390/ijms241210217 (PMC10299346; doi:10.3390/ijms241210217)
Supplement: Supplementary file 1 [file ijms-24-10217-s001.zip › Figure_S.pdf]

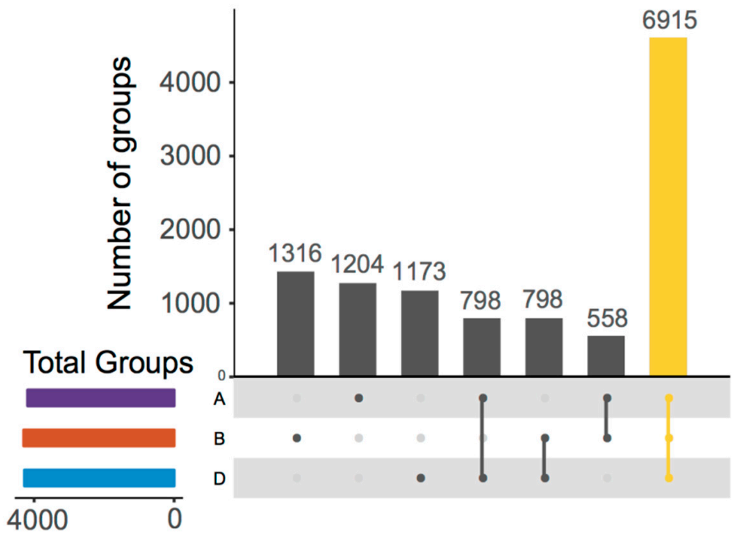

Figure S1. The paralogs classification in the AK58 genome.

Venn plot showing components and numbers of paralogous families in AK58 genome. Bars on the left indicate the number of paralogous families in subgenomes A, B and D, respectively. Bars on the top indicate number of IP, Dyad and Triplet families.

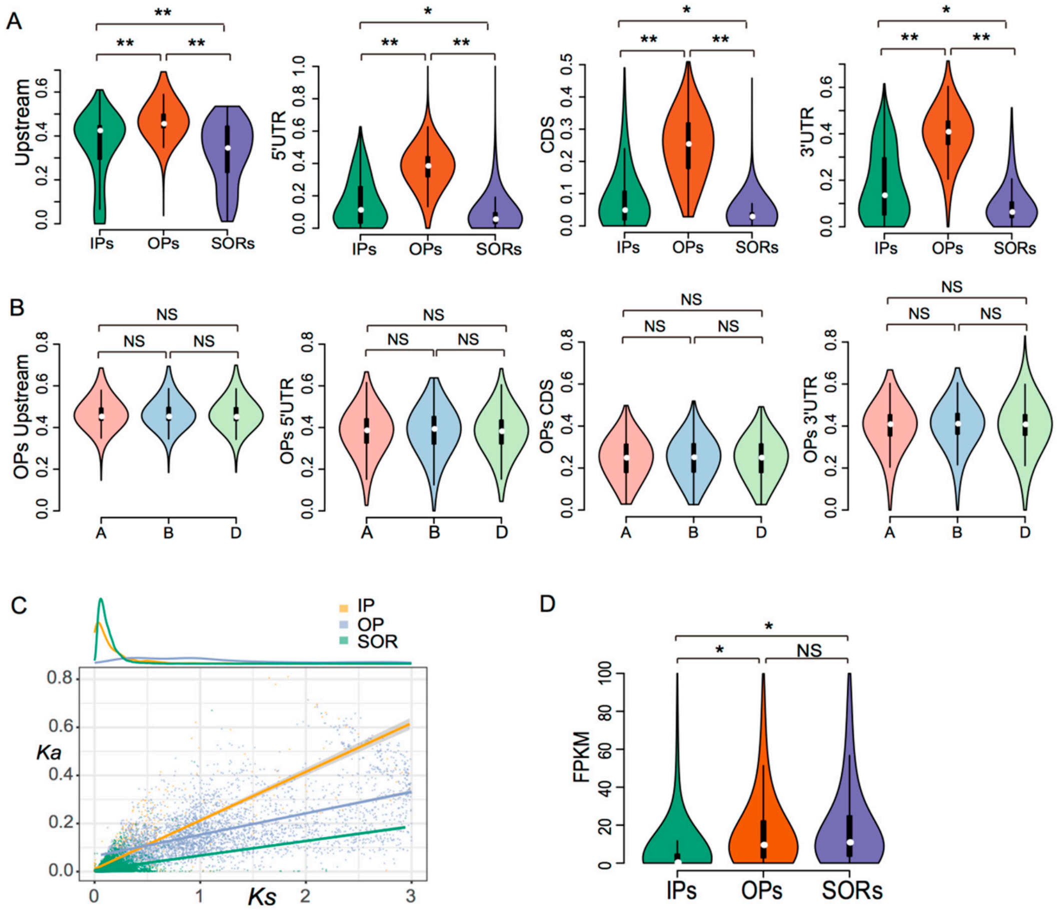

Figure S2. The nucleotide diversity and expression diversity of homologs.

(A) Sequence diversity of upstream 2Kb, 5'UTR, CDS and 3'UTR regions of homologs in Chinese Spring genome.

(B) Sequence diversity of upstream 2Kb, 5'UTR, CDS and 3'UTR regions of OPs in AK58 subgenomes A, B and D (NS= not significant).

(C) Dot plot showing *Ka* and *Ks* distribution of IPs, OPs and SORs in AK58 genome. The fit curves on the top show their *Ks* density for IPs, OPs and SORs.

(D) Expression levels (FPKM) of IPs, OPs and SORs in AK58 tissues.

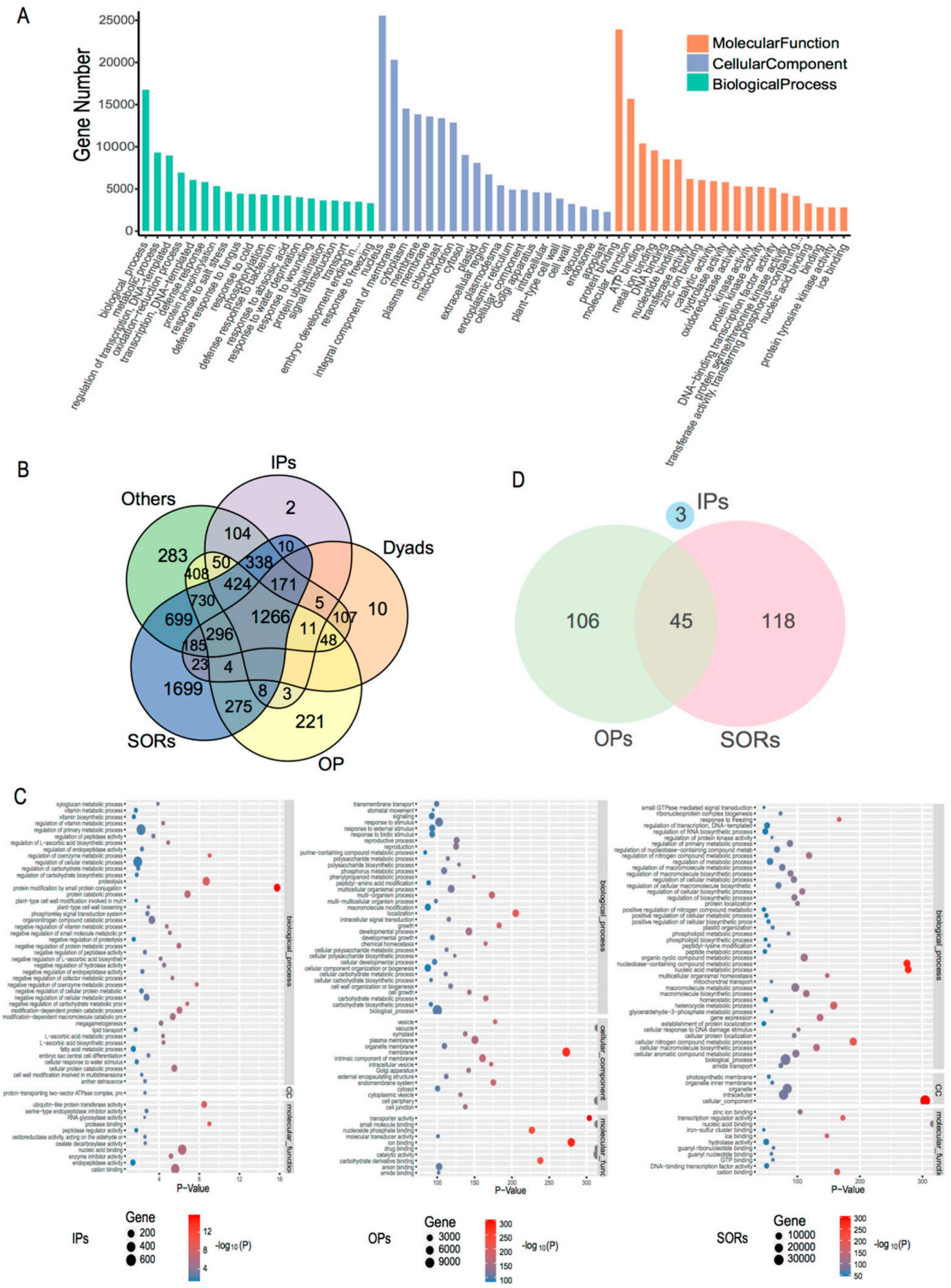

Figure S3. Functional diversity of AK58 homologs.

(A) Bar plot showing the most GO term annotations for all the AK58 HC genes.

(B) Numbers of annotated GO terms in different gene components. Genes that do not have distinct characteristics of IPs, Dyads, OPs, SORs are assigned as Others.

(C) Bubble plots showing the GO term enrichment analysis of IPs, OPs and SORs of AK58. The top 60 significantly enriched GO terms were presented in one plot for each type of homologs.

(D) Enriched PO terms specific to and shared by IPs, OPs and SORs. The figures in overlapping areas of circles indicate the number of shared PO terms, and non-overlapping areas are specific ones.

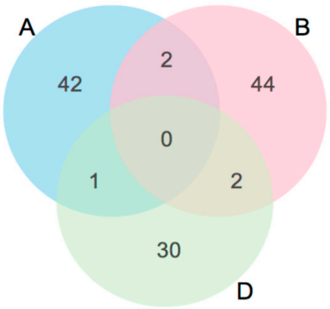

Figure S4. The KEGG divergence in AK58 wheat.

Enriched KEGG pathways specific to and shared by the subgenome specific WGDPGs in AK58. The figures in the overlapping areas of circles indicate the number of shared KEGG pathways, and non-overlapping areas are specific ones.

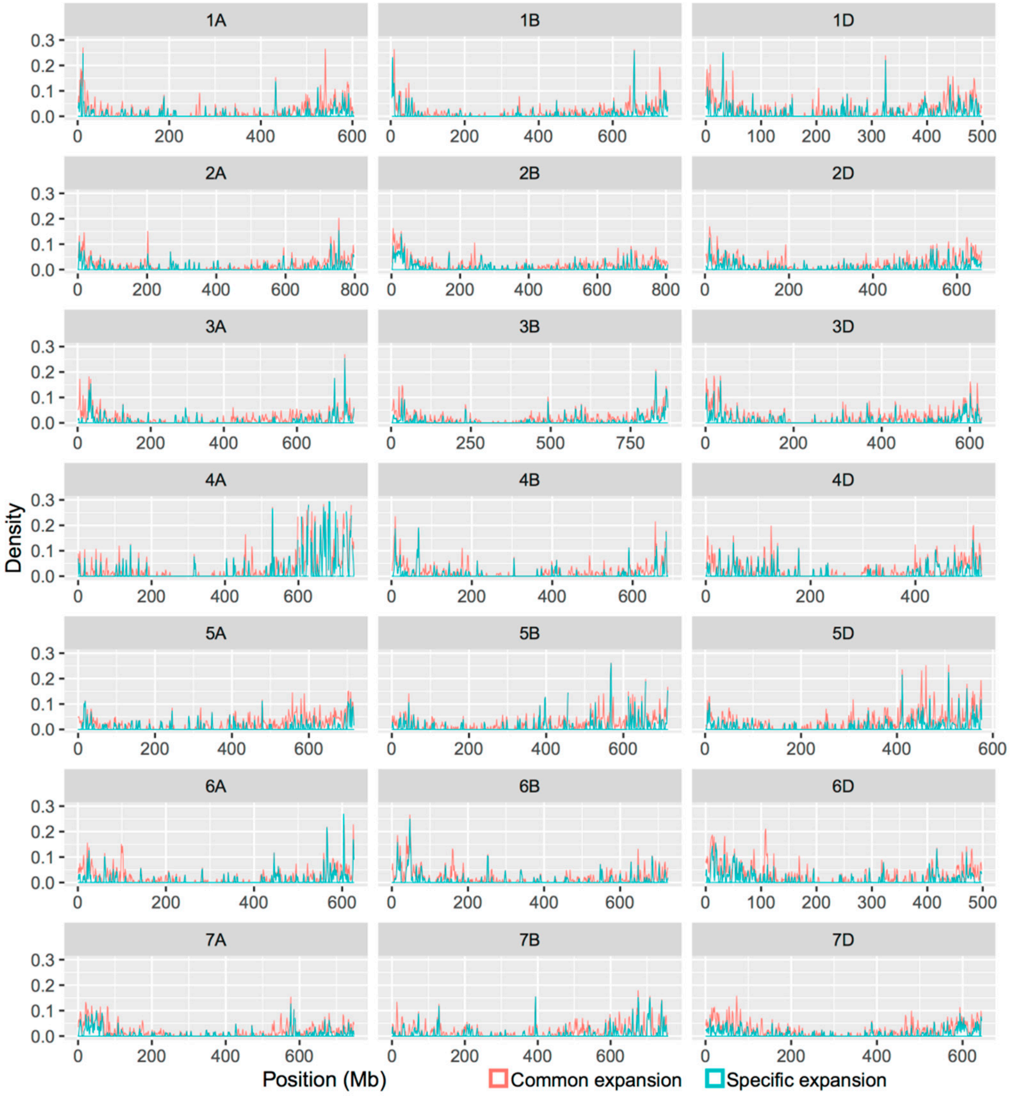

Figure S5. The density of common and specific expansion genes across the 21 chromosomes of AK58.

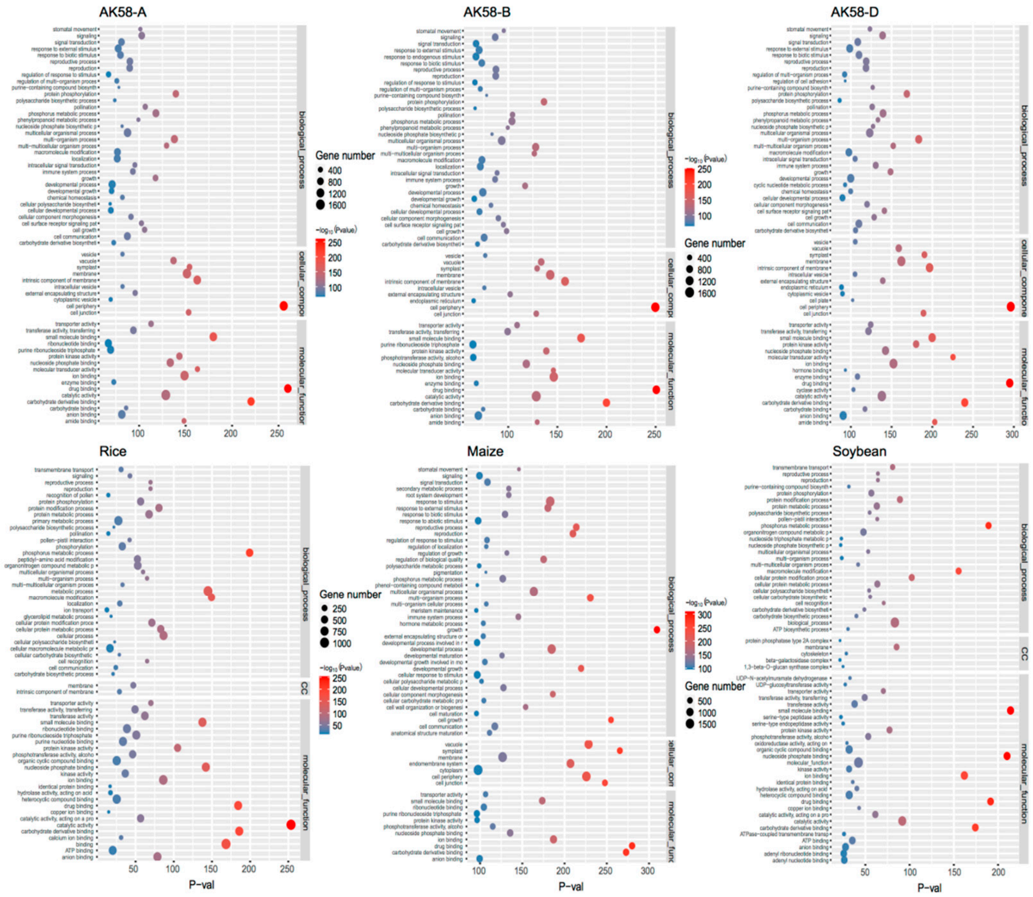

Figure S6. Enriched GO terms of common expansion genes in AK58, rice, maize and soybean genomes.

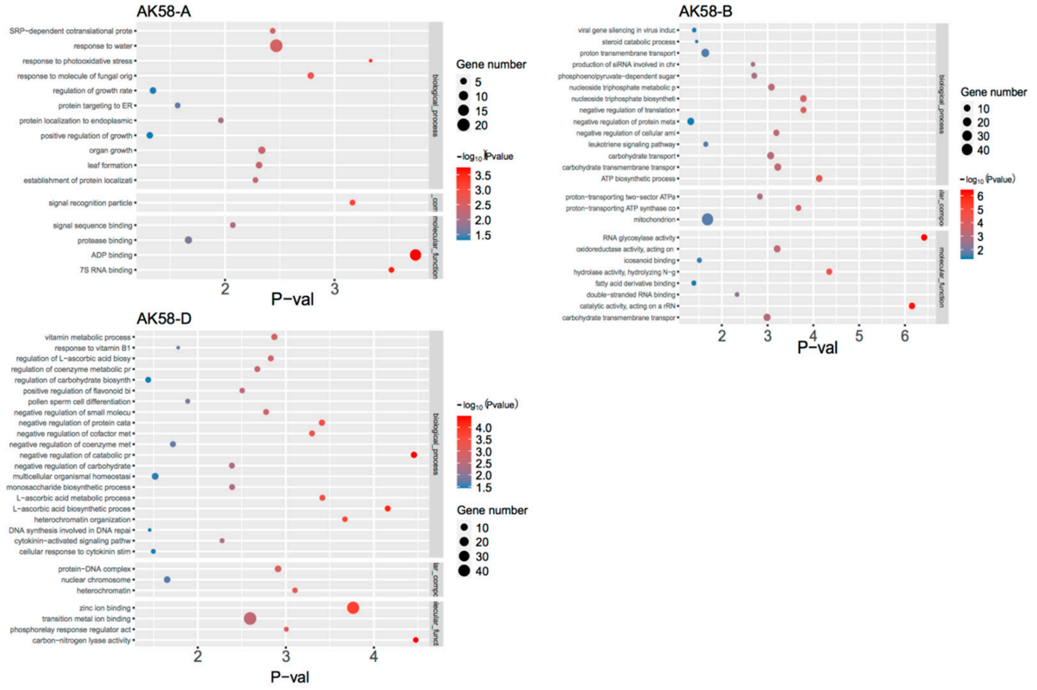

Figure S7. Enriched GO terms of specific expansion genes in subgenomes of AK58.

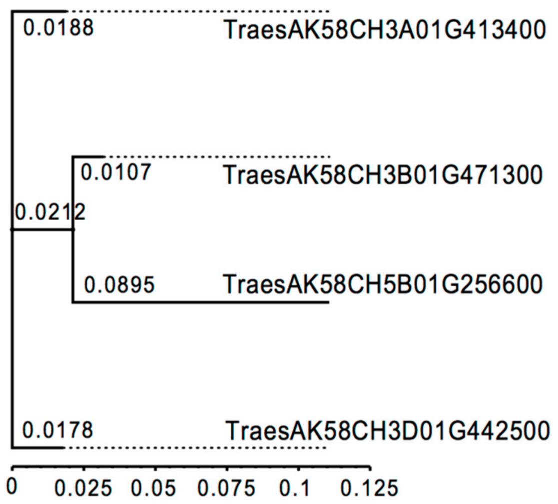

Figure S8. The phylogenetic tree of the *TaZIP4* family. Numbers represent the length of the branch where the leaf node is located. Phylogenetic tree was constructed by MEGA5 using the neighbor-joining and maximum-likelihood methods with 1000 replicates in the bootstrap test.

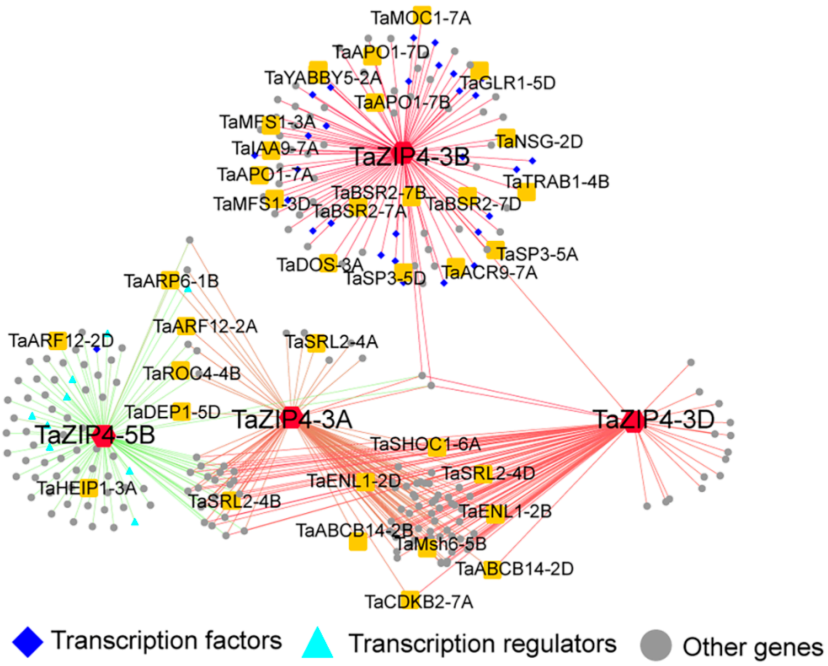

Figure S9. The co-expression network of *TaZIP4* and related genes.

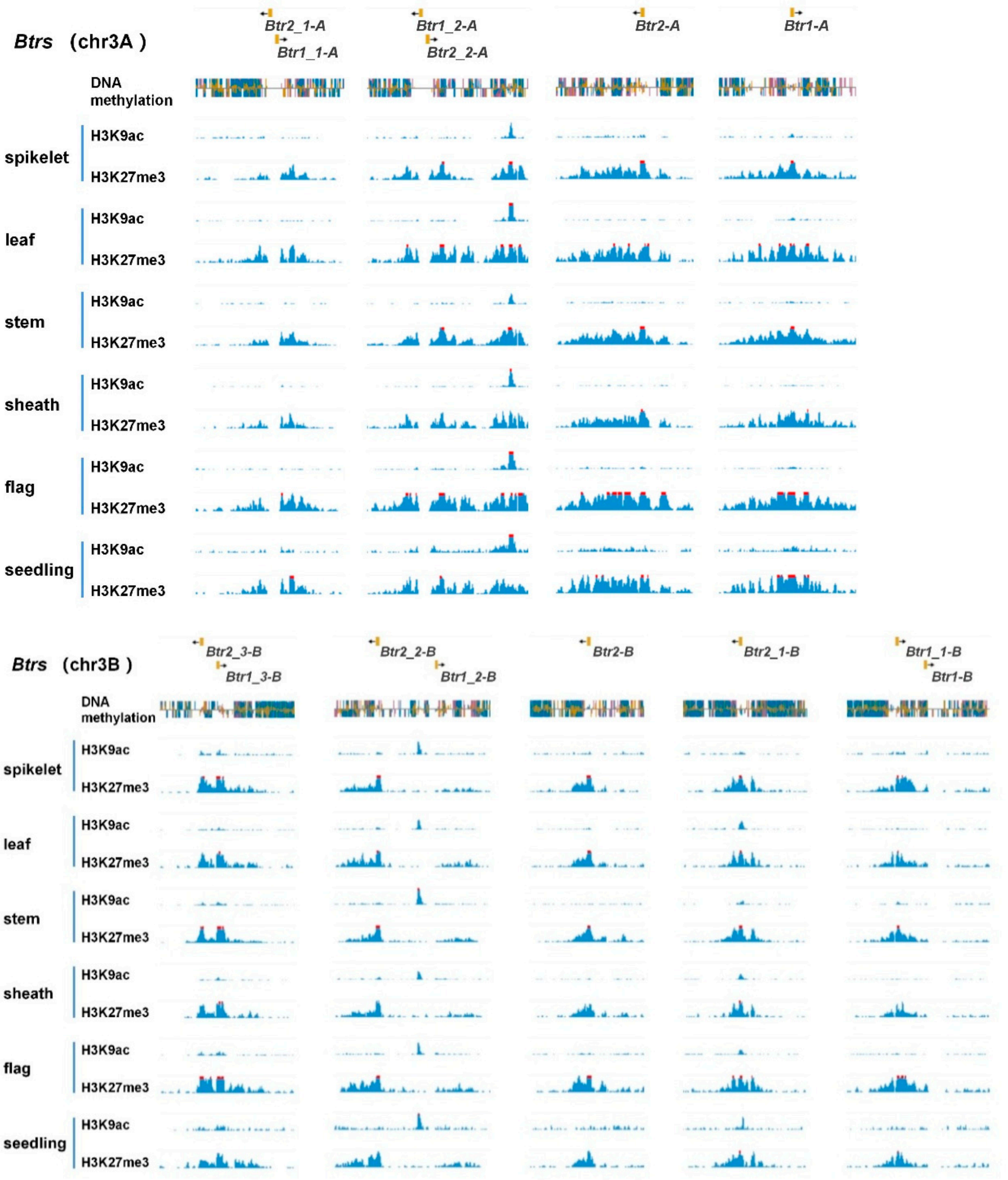

Figure S10. DNA methylation, H3K9ac and H3K27me3 modifications of *Btr* and *Btr-like* OPs in CS. The data are retrieved from Gene Expression Omnibus database (accession no. GSE139019). The gene diagrams are above the genomic tracks. The names are consistent with those in the wild emmer reported by Avni, Raz et al. (2017). In DNA methylation tracks, the purple sites indicate CG methylations, the blue sites indicate CHG methylations, and the orange sites indicates CHH methylations. The peak signals too high to be shown is truncated by red short lines.

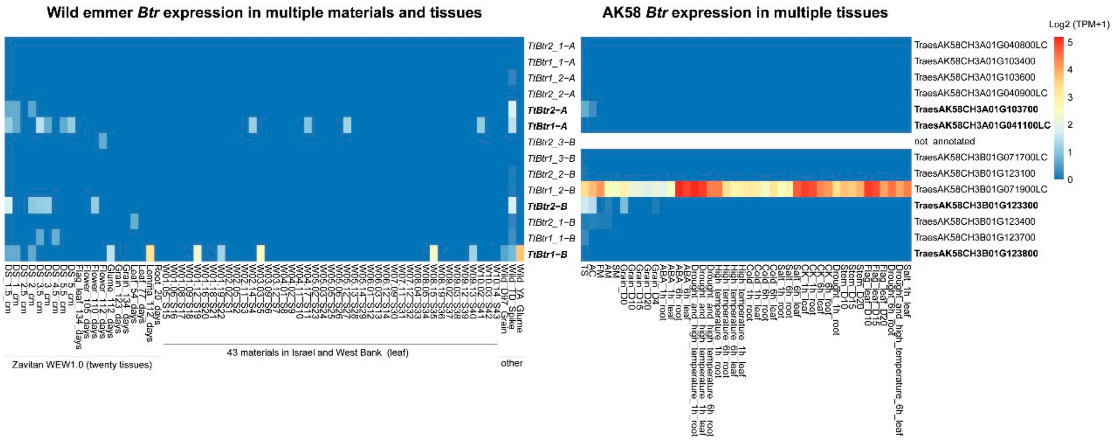

Figure S11. Gene expression (log2(TPM+1)) of *Btr* and *Btr-like* OPs in wild emmer and wheat cultivar AK58. The RNA-seq data of wild emmer are retrieved from European Nucleotide Archive (accession no. PRJEB19929) and NCBI BioProject (accession no. PRJNA507457, PRJNA272886, PRJNA288606, PRJNA777016).

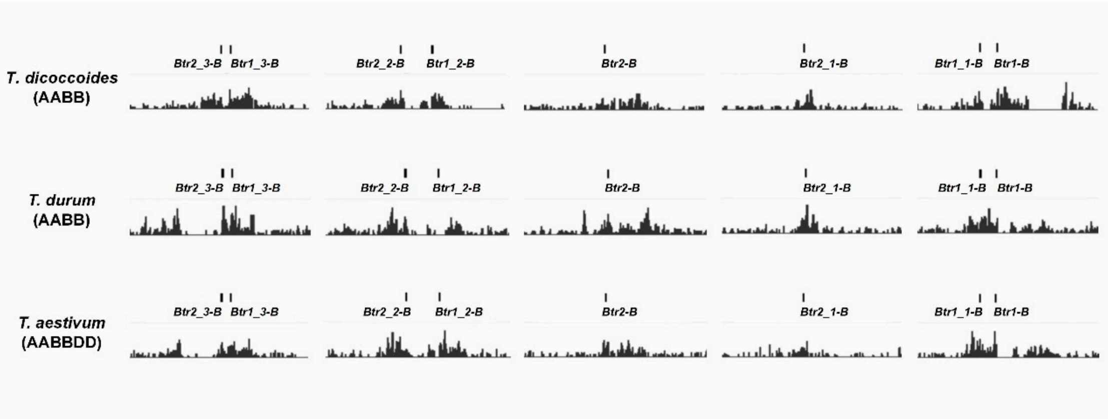

Figure S12. H3K27me3 modification of *Btr* and *Btr-like* OPs in *T. urartu* (AA), *T. dicoccoides* (AABB), *T. durum* (AABB) and *T. aestivum* (AABBDD) on chromosome 3B.
